# Implications of Nutritional Management on Fatty Acid Profiles of Southern White Rhinoceroses (*Ceratotherium simum simum*) Housed at Two Zoological Institutions

**DOI:** 10.3390/ani11113063

**Published:** 2021-10-27

**Authors:** Jordan Wood, Larry Jb Minter, Troy Neil Tollefson, Heidi Bissell, Doug Bibus, Kimberly Ange-van Heugten

**Affiliations:** 1 Department of Animal Science, North Carolina State University, Raleigh, NC 27695, USA; jnwood@ncsu.edu; 2 North Carolina Zoo, 4401 Zoo Pkwy, Asheboro, NC 27205, USA; jb.minter@nczoo.org; 3 Mazuri® Exotic Animal Nutrition, PMI Nutrition, Land O’Lakes, Inc., St. Louis, MO 63144, USA; TNTollefson@mzuri.com; 4 Busch Gardens Tampa Bay, 10165 McKinley Dr, Tampa, FL 33612, USA; Heidi.Bissell@seaworld.com; 5 Lipid Technologies LLC, P.O. Box 216, Austin, MN 55912, USA; doug@lipidlab.com

**Keywords:** fatty acid profiles, rhinoceros, whole blood

## Abstract

**Simple Summary:**

This study addresses the lack of information available for southern white rhinoceros circulating fatty acids that is important for the health, management, and conservation of the species. The objectives of this research were to provide novel fatty acid percentage profiles for managed healthy southern white rhinoceroses, as well as to provide comparisons between two zoos with differences in diet and climate during two distinct pasture growth periods. Noteworthy results were the higher levels of α-linolenic acid and total omega-3 fatty acids, and lower linoleic acid, total omega-6 fatty acids, and the omega-6 to omega-3 ratio found in Busch Gardens Tampa Bay in both growth periods when compared to the North Carolina Zoo. These data provide novel fatty acid percentages for southern white rhinoceroses and describe how fatty acids may differ between housing locations due to pasture growth periods and diet.

**Abstract:**

Southern white rhinoceroses (*Ceratotherium simum simum*) are African megaherbivores that are considered near threatened by the International Union for the Conservation of Nature. The fatty acid circulating values of these animals have not been thoroughly investigated. Fatty acids are critical for immune, heart, skin, and reproductive health, and may have a significant impact on the management and conservation of this species. Published data on fatty acids in this species is limited to incomplete profiles with very few animals in managed environments. The objectives of this research were to provide novel fatty acid percentage profiles for managed healthy southern white rhinoceroses, as well as to provide comparisons between two zoological institutions with differences in diet and climate during two distinct pasture growth periods. Whole blood samples were collected as dried blood spots from six rhinoceroses at the North Carolina Zoo (NC Zoo) and five rhinoceroses at Busch Gardens Tampa (BGT) in the low growth period (February to April) of 2019 and during the high growth period (July to September) of 2020. Fatty acid results indicated numerous differences when comparing the institutions within the same growth period and when comparing the same institution between its two growth periods. Most noteworthy were the higher levels of α-linolenic acid (18:3w3) and total omega-3 fatty acids and the lower linoleic acid (18:2w6), total omega-6 fatty acids, and omega-6 to omega-3 ratio found in the BGT population in both growth periods. This study provides novel percentages of fatty acids in managed southern white rhinoceroses and data on how fatty acid profiles may be altered between two housing locations via dietary differences in hay type and quantity, pasture availability via season, and pellet inclusion levels.

## 1. Introduction

Southern white rhinoceroses (*Ceratotherium simum simum*) are a primarily grazing species and are listed as Near Threatened by the IUCN [1]. Due to a continued decline in the free-ranging population due to increased poaching and habitat loss, it has been argued that a critically endangered classification is more appropriate [1]. Anti-poaching movements have been the cornerstone of free-ranging rhinoceros’ conservation, including the Southern White Rhinoceros Biodiversity Plan, which aimed to reduce poaching and increase population growth rates between 2015 and 2020 [2,3]. This plan was only partially successful with some increased population growth rates in areas around Kruger National Park, but ultimately did not reach the target population rates by 2020 [2]. Monitoring free-ranging population growth and anti-poaching strategies are only one part of total species conservation. To best conserve endangered species, such as southern white rhinoceroses, management of both free-ranging and human managed animals must be studied and better evaluated for insurance against extinction and education programs to enhance Species Survival Plans (SSP) [4]. Nutritional management of southern white rhinoceroses is one component of human care management that has not been explicitly examined with this species but is one of the five welfare domains under the 2021 Association of Zoos and Aquariums accreditation standards [4]. However, on-going work by the authors and their collaborators have focused on the role of nutrition on blood nutrient parameters, including fatty acids in similar African megaherbivore species [5,6].

Fatty acids directly impact numerous physiological functions including immune status and reproductive success [7], which have a significant impact on institutional animal management and conservation efforts. Southern white rhinoceroses under managed care often struggle with obesity and subfertility due to diet [8,9]. In mammals, the quantity of dietary essential omega-3 and omega-6 polyunsaturated fatty acids (PUFA) can influence prostaglandin production thus altering ovulation rates, progesterone production, gestation length, post-partum body condition, offspring weight and survival, as well as spermatozoa structure and mobility [10,11,12,13]. Research in many exotic species demonstrates that managed animals have different fatty acid profiles compared to their free-ranging counterparts, generally reflected in lower percentages of PUFA and the dietary essential omega-3 fatty acids in human managed animals [14,15]. As omega-3 and omega-6 PUFA share the same enzymes for desaturation and elongation, an overabundance of either group may be limiting and therefore have clinical significance to managed animals due to their effects on reproductive success, amongst other health concerns. Dietary fatty acid profile can often be modified by changing dietary inputs [16,17]. Seasonal shifts in fatty acids related to dietary management have been seen in both human managed domestic and exotic herbivores [6,18]. Changes in seasonal data were often correlated with reduced access to grazing material during the colder months and shifts to include more dry hay and pelleted feed items [6,18]. Thus, knowledge of the fatty acid profiles of animals at multiple institutions across two seasons, especially those with climate differences, can forecast the need for alterations in the management and feeding of human managed animals.

Alternative methods for fatty acid analysis provide information about fatty acid status of an animal in one drop of dried whole blood placed on filter paper cards [19,20,21]. Whole blood provides more accurate information about long term fatty acid status as compared to plasma or serum, which generally reflect short term fatty acid status [22,23]. The small volume of blood required with dried blood spot (DBS) samples improves the ability to examine blood for fatty acid profiles as analyses can be performed using multiple DBS cards to ensure the percentages are the best representation of fatty acids present [24]. The use of DBS cards also makes collections much more feasible, as it does not require immediate freezing or refrigeration and can be transported easily [24].

The purpose of this research is threefold: (1) to provide novel whole blood fatty acid percentage profiles for managed southern white rhinoceroses, (2) to compare the fatty acid profiles of southern white rhinoceroses at two zoological institutions with different diets, and (3) to identify if unique locational pasture growth period changes alter fatty acid percentages between the two institutions and between the two growth periods within each institution.

## 2. Materials and Methods

### 2.1. Animals, Sample Collection, and Analysis

This study was approved by the NC Zoo Animal Research Committee and the Busch Gardens Tampa Research Committee. Adult southern white rhinoceroses were bled via medial radial veins or an auricular vein in untreated red top serum separator tubes (BD vacutainer, Franklin Lakes, NJ, USA) as whole blood via animal cooperation (no physical or chemical restraints) at the North Carolina (NC) Zoo in Asheboro, NC, USA (*n* = 6, 1 male and 5 females) in February 2019 and July/August 2020 and Busch Gardens Tampa Bay (BGT) in Tampa, FL, USA (*n* = 5, 1 male and 4 females) in April 2019 and September 2020. Samples were immediately transferred to two PerkinElmer 226 Spot Saver dried blood spot filter cards (DBS card) per animal with a needle and syringe before air drying. Each spot on a DBS card can hold approximately 80 μL of whole blood for a total of 320 μL of whole blood per card or 640 μL per animal. After collection and drying, DBS cards were stored at approximately room temperature (23 ℃) before being transferred to NC State University (Raleigh, NC, USA, 27695) for 1 week for shipment to Lipid Technologies for a whole blood fatty acid panel including 36 individual fatty acids and 10 fatty acid groups (Table 1) utilizing proprietary methods (Lipid Technologies, Austin, MN, USA 55912; https://lipidlab.com/services/ (accessed on 25 October 2021)). This method employs transmethylation with acidified methanol, then fatty acid methyl esters, before being analyzed by gas chromatography [25].

Southern white rhinoceroses managed at the NC Zoo have access to a large exhibit with exhibit grasses year-round and are provided 1.4 kg of Mazuri^®^ Wild Herbivore Hi Fiber pellets (St. Louis, MO, USA (Mazuri^®^ Wild Herbivore Hi-Fiber Diet (purinamills.com))) (accessed on 25 October 2021). per rhinoceros on a daily basis. During the warmer seasons/high pasture growth periods of summer and fall, two bales of timothy hay (*Phleum pratense*) were available to the entire herd (six adults, two calves) during the day, and this increased to a bale for each animal in the colder seasons/low pasture growth periods of winter and spring along with supplemental timothy hay cubes for training purposes. Exhibit grasses were previously nutritionally characterized [5] and include: fescue (*Festuca arundinacea*), Bermuda (*Cynodon dactylon*), and annual ryegrass (*Lolium multiflorum*). The diet of the animals at BGT was not nutritionally analyzed. However, it did not include any nutritionally complete pellet and consisted of three quarters of a bale of timothy hay per animal and free-grazing access on an exhibit of mixed golf course Bermuda grass during the day. The rhinoceroses at BGT were not reported to consume any pelleted feeds intended for other species maintained in their mixed species exhibit. Low pasture growth periods were defined as February, 2019 for NC and April, 2019 for BGT while high pasture growth periods were defined as July/August 2020 for NC and September 2020 for BGT.

### 2.2. Statistical Analysis

Fatty acid percentage means were calculated based on four primary groupings: two pasture growth periods (low growth and high growth) and two locations (NC Zoo (*n* = 6) and BGT (*n* = 5)). Statistical comparisons were conducted using the Kruskal–Wallis *H*-Test with pairwise comparisons using the Mann–Whitney *U*-Test with an α = 0.05 to determine if any individual fatty acids or groups differed between: (1) low growth period samples collected at the NC Zoo and BGT, (2) high growth period samples collected at the NC Zoo and BGT, (3) low growth and high growth period samples collected at the NC Zoo, and (4) low growth and high growth period samples collected at BGT. Statistical analysis was performed using IBM SPSS Statistics for Windows (Version 25.0 IBM Corp. Armonk, NY, USA) and results were verified by performing the same tests through written calculations.

## 3. Results

All southern white rhinoceroses maintained good health throughout the sampling period and throughout the time between samples. The entire herd at BGT maintained a body condition score (BCS) of a six on a nine-point scale throughout 2019 and 2020. At the NC Zoo, one rhinoceros remained at a seven, one remained at a six, and two remained at a five throughout 2019 and 2020. Two of the females were pregnant in 2019 and each had a BCS of six which decreased to a five in 2020 for both females after parturition. At the time of the 2020 sample collection, both of these females were lactating but diets remained the same.

Of the 36 individual fatty acids analyzed, 26 were present in large enough quantities to be identified in the DBS samples (Table 1). The 10 fatty acids that were not quantified were lauric acid (12:0), myristoleic acid (14:1), pentadecylic acid (15:0), pentadecanoic acid (15:1), 9-hexadecenoic acid (16:1w5), margaric acid (17:0), heptadecenoic acid (17:1), 13-octadecenoic acid (18:1w5), vaccenic acid (18:1w7), and eicosenoic acid (20:1w9). All 10 fatty acid groups were identified in the samples.

Eight individual fatty acids, including stearic acid (18:0), oleic acid (18:1w9), α-linolenic acid (18:3w3), arachdic acid (20:0), paullinic acid (20:1w7), eicosatrienoic acid (20:3w3), eicosatetraenoic acid (20:4w3), and omega-6 docosapentaenoic acid (DPA) (22:5w6), were higher in percentages in the southern white rhinoceroses managed at BGT compared to the NC Zoo when comparing the two low pasture growth. Total saturates, total monoenes, total omega-3s, total omega-9s, and total omega-3 HUFAs were also higher in the BGT animals than in the NC Zoo animals in the low growth period. Three individual fatty acids, linoleic acid (18:2w6), γ-linolenic acid (18:3w6), and mead acid (20:3w9), were higher in the low growth samples from the NC Zoo southern white rhinoceroses than the BGT rhinoceroses. Total PUFAs, total omega-6s, total omega-6 HUFAs, and the omega-6 to omega-3 ratios were also higher in the NC Zoo animals than the BGT animals in the low pasture growth period.

In contrast, more individual fatty acids were higher in the NC Zoo rhinoceroses than in the BGT rhinoceroses when comparing samples collected in the high growth period at both locations. The nine higher fatty acids and four fatty acid groups included: linoleic acid (18:2w6), arachdic acid (20:0), h-γ-linolenic acid (20:3w6), arachidonic acid (20:4w6), eicosapentaenoic acid (20:5w3), behenic acid (22:0), docosatetraenoic acid (22:4w6), lignoceric acid (24:0), and docosahexaenoic acid (DHA) (22:6w3), total PUFAs, total HUFAs, total omega-6s, and the omega-6:omega-3 ratio. Three individual fatty acids, oleic acid (18:1w9), α-linolenic acid (18:3w3), and eicosatrienoic acid (20:3w3), and five fatty acid groups, total monoenes, total omega-3s, total omega-9s, total omega-3 HUFAs, and total omega-6 HUFAs, were found in lower percentages in the high pasture growth samples collected at the NC Zoo when compared to the high pasture growth samples from BGT.

When comparing growth periods within each zoo location, a total of 12 fatty individual acids and two fatty acid groups had pasture growth time period differences at NC Zoo, while ten individual fatty acids and no fatty acid groups had growth period differences at BGT. At the NC Zoo, low pasture growth DBS cards had lower percentages of stearidonic acid (18:4w3), arachdic acid (20:0), paullinic acid (20:1w7), h-γ-linolenic acid (20:3w6), arachidonic acid (20:4w6), eicosapentaenoic acid (EPA) (20:5w3), erucic acid (22:1w9), docosatetraenoic acid (22:4w6), omega-3 DPA (22:5w3), lignoceric acid (24:0), nervonic acid (24:1), total saturates, and total HUFAs than high pasture growth time period samples, while only eicosatetraenoic acid (20:4w3) was higher in the high growth period. Stearidonic acid (18:4w3), γ-linolenic acid (18:3w6), paullinic acid (20:1w7), mead acid (20:3w9), erucic acid (22:1w9), omega-3 DPA (22:5w3), and lignoceric acid (24:0) had lower percentages in low pasture growth time period samples than in the high pasture growth samples at BGT. Eicosatetraenoic acid (20:4w3), omega-6 DPA (22:5w6), and docosahexaenoic acid (DHA) (22:6w3) were both higher in the low growth samples than in the high pasture growth samples collected at BGT.

## 4. Discussion

To date, this data represents the largest novel dataset of fatty acids in southern white rhinoceroses. Results found variations in α-linolenic acid, linoleic acid, total omega-3s, total omega-6s, total PUFAs, total omega-3 HUFAs, total omega-6 HUFAs, and the omega-6:omega-3 ratio when comparing institutions within each growth period. In general, omega-3 fatty acids were higher in BGT rhinoceroses in both low and high growth periods. When comparing growth period within zoo location, the essential fatty acids and omega fatty acid groups were similar between the two growth periods.

The most well nutritionally studied species of grazing herbivore that is potentially similar to southern white rhinoceroses is the domestic horse (*Equus caballus*). In a study analyzing the serum fatty acid profile in the domestic horse, there were ten analogous individual fatty acids and five analogous fatty acid grounds which we also analyzed [26]. A majority of the fatty acids analyzed, including palmitic acid (19.8%), stearic acid (29.9%), linoleic acid (34.0%), stearidonic acid (0.20%), h-γ-linolenic acid (0.74%), eicosatetraenoic acid (0.25%), docosahexaenoic acid (0.94%), total saturates (44.9%), PUFAs (38.9%), and omega-6 PUFAs (36.2%), were lower in the southern white rhinoceroses than in horses [26]. The only fatty acid that was similar between species was omega-3 DPA (0.24%) as the other four fatty acids and groups, α-linolenic acid (1.0%), arachidonic acid (1.3%), total monoenes (13.1%), and omega-3 PUFAs (1.7%), were higher in southern white rhinoceroses than in horses [26]. These values depict significant variation between these two species that can be attributed to species differences as well as dietary management differences. The horses in [26] were fed meadow hay and barley grain which could attribute to the higher omega-6 values found in circulation. Comparatively, the southern white rhinoceroses are raised primarily on pasture and hay which is higher in omega-3 fatty acids. It is difficult to directly compare the current data with much of the available horse literature, as horses are often fed a more complex diet and most research performed does not analyze for free fatty acids or does not analyze free fatty acids on a percentage basis.

To date, there are very few data available on circulating fatty acids in free-ranging or managed southern white rhinoceroses. The data that are available come from free fatty acids or lipid fractions in serum, as opposed to whole blood or DBS, and include incomplete profiles [27]. Work to validate the use of DBS for fatty acid analysis and monitoring in megaherbivores, including southern white rhinoceroses, is still required, but is a part of on-going work by the current investigators. Unpublished data by the primary investigator and co-authors have evaluated fatty acids from DBS, whole blood, serum, and plasma samples collected from a similar African megaherbivore species, the African savanna elephant (*Loxodonta africana*) [28]. Current results of this work have found that there are very few differences between sample types, with most differences being in nonessential and trace fatty acids, offering evidence that compares across sample types may be possible when comparing free fatty acid percentages out of the total fatty acid percentage. Additionally, there are limited full fatty acid profiles for other rhinoceros’ species with only serum percentages available for greater one-horned rhinoceroses (*Rhinoceros unicornis*) [29] and for black rhinoceroses (*Diceros bicornis*) [27]. Values available for managed white rhinoceroses (*n* = 4) are divided by serum/plasma triglyceride, phospholipid, and cholesterol ester fractions on a percent of total fatty acid basis [27]. For the purposes of comparisons to the data from this current study, ranges across all three fractions were used to build a percent range (Supplementary Table S1). Compared to the current study, [27] only quantified eight individual fatty acids and five groups that could be matched. The averages of all growth periods found at the NC Zoo and BGT were within the ranges found by [27] for palmitic acid (10.9–43.3%), stearic acid (1.5–30.8%), linoleic acid (11.6–67.3%), total saturates (13.6–54.1%), and PUFAs (13.6–69.3%), though most were on the lower end of the ranges found by [27]. The averages found at the NC Zoo and BGT were higher than the ranges found by [27] for oleic acid (9.5–22.9%), α-linolenic acid (0.2–1.0%), arachidonic acid (0.0–1.3%), total monoenes (12.3–29.2%), total omega-6 fatty acids (12.6–68.3%), and total omega-3 fatty acids (0.2–1.0%). As there were only four animals used in [27], and the values were based on multiple fractions, it is difficult to say if being on the lower end of these ranges or being above these ranges is positive or negative for the animals in the current study. It was difficult to relate the omega-6 to omega-3 ratio in the current study to the prior work, as the previous work provided the omega-3 to omega-6 ratio instead [27]. By taking the averages found for total omega-6 and omega-3 fatty acids from the NC Zoo and BGT, a ratio of the omega-3 to omega-6 fatty acids was built as a substitute. Comparing these new values to the ranges found by [27], it was found that the omega-3 to omega-6 ratio was higher in the current study. These ratios are important, as omega-3 and omega-6 PUFAs share the same enzymes for desaturation and elongation into important PUFAs. An overabundance of either group may be limiting for specific PUFAs, and therefore have clinical significance to managed animals due to their effects on health.

When evaluating these white rhinoceros ranges, it is important to consider that these are not perfect comparisons due to the separate fractions within the serum/plasma as opposed to DBS samples, and that the samples from [27] are not specified as northern or southern white rhinoceroses. Regardless, the values seen suggest that the animals housed at the NC Zoo and BGT likely have similar values to those collected by [27] from other managed rhinoceroses within North America. It also shows that over recent years both total omega-6 and omega-3 fatty acids may have increased in white rhinoceroses in managed care. These differences could be due to the disparities in methodology and analysis, but could also be attributed to alterations in diets of these animals. Many institutions have balanced their diets to allow for a decrease in needed nutritionally complete pellet inclusion levels, and manufacturers have altered formulations of nutritionally complete pellets to decrease the omega-6 content. These alterations would allow for a better forage to pellet ratio and lead to higher omega-3 values. Regardless, due to the sparsity of data for this species, the authors are excited to provide the extensive fatty acid data in this publication for future subsequent evaluations.

The circulating levels of omega-3 fatty acids were higher in the BGT animals that were being fed strictly forages in the low pasture growth period. Conversely, the circulating levels of omega-6 fatty acids were higher in the NC Zoo animals that were being provided forages and pellets in the low growth period. Additionally, the ratio of omega-6 fatty acids to omega-3 fatty acids was higher in the NC Zoo animals than the BGT animals during the low growth period. The diets of the NC Zoo and BGT differ in a few ways such as the inclusion of a small percentage of commercial nutritionally complete pellet in the diet of the NC Zoo animals, the amount of hay provided during different time periods, and the type of pasture grass that is grazed. While the NC Zoo provides their rhinoceroses with 1.4 kg of Mazuri^®^ Wild Herbivore pellets daily, BGT does not provide any pellets throughout the year. Additionally, the NC Zoo rhinoceroses are provided additional timothy hay in the colder months to supplement for the decreased grass availability. NC Zoo does experience much more seasonal temperature extremes than BGT does.

To further examine the dietary factors in the noted circulating differences, samples were collected from the same animals at both locations during the high pasture growth period of 2020, when the NC Zoo was feeding additional timothy hay and timothy cubes while still feeding Mazuri^®^ Wild Herbivore pellets. Results from the comparison of these two locations for each growth period found that percentages of omega-3 fatty acids were still lower in the NC Zoo rhinoceroses than in the BGT rhinoceroses in the high growth period, and that the omega-6 to omega-3 ratio and total omega-6 fatty acids were also consistent for the high and low growth periods with a higher ratio at the NC Zoo. With these dietary differences, the fatty acid results showed differences in total omega-3 and total omega-6 fatty acids between the two groups of animals that are consistent with trends noted in the literature [30,31,32]. The pellets fed at the NC Zoo are also low in omega-6 fatty acids (approximately 1.35%). These consistent trends indicate that the differences between these two locations goes beyond the inclusion of pellets in the diet and is also associated with the differences in forages, including pasture and hay, available to these animals. The greater pasture availability found at BGT may be a benefit to the diet, but this is still a component of the diet that needs to be better examined.

Relative fatty acid content data on the hay and grass available to the NC Zoo rhinoceros herd was analyzed during a study performed between 2016 and 2017 with the neighboring African elephant herd [33]. The pasture values collected during this study found an average of 54.3% omega-3 fatty acids and 10.3% omega-6 fatty acids for a comparable low growth period and 44.7% omega-3 fatty acids and 14.4% omega-6 fatty acids for a comparable high growth period [33]. The timothy hay values collected found an average of 26.3% omega-3 fatty acids and 18.3% omega-6 fatty acids in the low growth period and similar values (30.3% and 16.7%, respectively) in the high growth period [33]. Considering the increase in hay intake coupled with the decrease in pasture intake during the low growth period for the NC Zoo, it is not surprising that the total omega-6 fatty acids were higher when compared to BGT as the hay would increase omega-6 intake. The lack of statistical differences in the omega-6 to omega-3 ratio and the total omega-6 fatty acids between low growth and high growth collections at the NC Zoo or at BGT provides additional evidence for this dietary hypothesis. Currently, no fatty acid analysis of the hay or pasture has been conducted at BGT nor surrounding facilities within Florida to compare to the NC Zoo.

Higher omega-6 fatty acids are found in managed exotic herbivore diets with more hay and pelleted feeds than those of free-ranging herbivores that have forage-based diets which typically contain higher levels of omega-3 fatty acids [30,31,32]. These dietary fatty acids have a direct correlation with the circulating levels of these fatty acid groups and, in managed exotic herbivores, can lead to imbalanced omega-6 to omega-3 ratios [30]. Data available from free-ranging black rhinoceroses support the general observations previously demonstrated of higher levels of omega-3 fatty acids in free-ranging versus managed animals [17,27]. This is not surprising, as diet items consumed in the wild, including browse and grass, generally contain higher omega-3 fatty acid content as compared to traditional zoo diets [32,34,35]. While some literature is available with data comparing free-ranging and managed rhinoceros species, additional research is encouraged, especially in southern white rhinoceroses. Fatty acid profiles can be modified by diet. For example, the addition of a flaxseed-based supplement (rich in 18:3 linolenic acid) numerically increased 18:3 levels in the blood of black rhinoceroses [17]. Work in horses, which have a similar gastrointestinal tract to rhinoceroses, has also demonstrated the ability to modify plasma, red blood cell, and skeletal muscle fatty acid profiles with diet [36,37]. The inclusion of pelleted feed in the diet of southern white rhinoceroses is only recommended when the energy requirements of the animals are not being met by the amounts of forages that are available [38,39]. Due to the growth period variability of available grasses on exhibit at the NC Zoo, the zoo feeds additional timothy hay and hay cubes alongside the pelleted diet to provide appropriate dietary energy, a diet shift that is not necessary in Florida due to the warmer temperatures year-round and the subsequent longer growing season. We note that a limitation of our study is that our blood samples were not collected during the same month for the low or high pasture growth periods at each zoo. This limitation was related to both travel and COVID-19 logistics. While we do not believe the small time difference between sample dates made a large impact on our data, we do acknowledge that the seasonality variations on forages between Asheboro, NC and Tampa, FL may complicate interpretation.

It is also important to note that the inclusion of pellets in the NC Zoo diet may not be a main source for meeting fatty acid requirements due to natural physiological adaptations in southern white rhinoceroses. In free-ranging southern white rhinoceroses, it has been seen that the animals will graze extensively during the wet season and develop fat reserves during this time, which can be used in the dry season when the ability to graze is greatly reduced [40,41,42]. While this is a physiological adaptation to decreased feed, all animals in the study maintained a desirable BCS within a five to a seven on a nine-point scale when fed or supplemented year-round. This maintenance of BCS could be due to a change in adaptations for managed animals, or could be due to the high grazing activity of these animals during the year. Additionally, both institutions have highly successful breeding programs and have had several females cycling through pregnancy and lactation which can alter fatty acid mobilization and BCS scores. Due to this natural response to less grazing availability, if the pasture intake during the rest of the year remains high the rhinoceroses maintained at the NC Zoo may have fat reserves that will provide sufficient energy, maintain appropriate body weight, and appropriate fatty acid percentages without the inclusion of large amounts of pellets (beyond 1–3% of body weight) in the diet. With the small amount of pellets fed at the NC Zoo (below 3% of body weight), this is most likely how the animals are maintaining appropriate weights and BCS while having successful reproductive status.

Both parent essential fatty acids (EFA) from which the other essential PUFAs can be metabolized, linoleic acid (18:2w6) for omega 6 PUFAs and α-linolenic acid (18:3w3) for omega 3 PUFAs, had differences when comparing the two locations within low and high pasture growth periods. The levels of α-linolenic acid were higher in the BGT rhinoceroses than the NC Zoo rhinoceroses in both the low and high growth periods. Conversely, the levels of linoleic acid were lower in the BGT animals compared to the NC Zoo animals in both growth periods. These two fatty acids line up with the significant differences found between total omega-3 and omega-6 fatty acids for each of these institutions, suggesting these could be the primary omega-3 and omega-6 fatty acids present in circulation. These differences could also be attributed to the increase in pasture intake between these two locations at the time of blood sampling. Due to the more consistently warm temperatures in Florida, pasture grass could have been lusher at BGT than the similar growth period at the NC Zoo, which would alter the circulating omega-3 and omega-6 percentages. The primary role of linoleic acid as an EFA is to act as a precursor for long chain fatty acids such as DHA and EPA, but it can also impact skin and hair health, growth rates, cognitive abilities, and immunity [43]. A higher percentage of α-linolenic acid is important for cardiovascular health and decreasing obesity concerns as it is a precursor for docosahexaenoic acid (DHA) and eicosapentaenoic acid, which impact blood pressure, heart rate, and the prevalence of arrhythmias (EPA) [44,45]. In humans, α-linolenic acid has an inverse relationship with adipose tissue development [45]. The concern about high levels of adipose tissue development and obesity is not limited to humans; many exotic species can also suffer from obesity, leading to body conditioning and weight being a serious concern [9]. In another African megaherbivore species, the African elephant (*Loxodonta africana*), α-linolenic acid is critical for spermatozoa development in males, and dietary deficiencies can negatively impact reproductive success [46]. Due to the similarities in dietary management and GI morphology of these two species, it can be inferred that southern white rhinoceros males could have a similar response to α-linolenic acid. In females of various species, it has been found that supplemental PUFAs can positively impact prostaglandin synthesis and increase the mass and body condition of the mother and her offspring, thus positively influencing the energy needs of lactation [10,11,12]. The NC Zoo and BGT have had significant success with their rhinoceros breeding program, and while we cannot directly link fatty acids to the reproductive success of these populations, it is interesting, and the authors believe that knowing these successful, yet slightly different, percentages for reproductively successful animals is important for future population comparisons.

The lack of literature available in whole blood or southern white rhinoceroses makes the current data set valuable as the beginning parameters for whole blood fatty acids in managed southern white rhinoceroses and provides one of the largest samples of successfully managed southern white rhinoceroses with both dietary and pasture growth period changes noted. Having data with two institutions, two diets, and two represented growth periods can provide invaluable information to better manage this species in a variety of locations. However, this is only the preliminary data for DBS fatty acid values in southern white rhinoceroses and should not be considered a true reference range or average as the total sample size was only 12 animals. Further research and sampling is recommended to continue developing an accurate reference range of DBS fatty acids for southern white rhinoceroses. Understanding the appropriate levels of circulating fatty acids in whole blood would be improved by having samples from not only managed southern white rhinoceroses but also free-ranging animals. This comparison would provide further information on the long-term fatty acid status of animals in their natural habitat that can influence the dietary management of fatty acids in zoological institutions.

## 5. Conclusions

This data provides a novel and a complete profile for circulating fatty acids in the less studied southern white rhinoceros species while offering further comparison between two dietary management plans and climate zones. Further sample collection and research is recommended to nutritionally compare the exact diets of these two locations during the same pasture growth periods to determine if the omega-3 and omega-6 fatty acid differences are due to forage differences, pellet inclusion, or other factors. The current data provides insight into how dietary management of fatty acids can positively influence the overall health, cardiovascular health, and weight in managed animals and could influence the success of reproductive programs at zoos and the conservation of the species as a whole.

## Figures and Tables

**Table 1:** Fatty Acid (%) Profile Averages and Standard Deviations (SD) of NC Zoo Dry Blood Spot (DBS) Samples (*n* = 6) and Busch Gardens Tampa DBS Samples (*n* = 5) from Managed Southern White Rhinoceroses (*Ceratotherium simum simum*) during both Low and High Pasture Growth Periods with a Mann–Whitney *U*-Test Pairwise Comparisons ^1^.

Individual Fatty Acid	North Carolina Zoo		Busch Gardens Tampa	
	Low Growth Average (SD)	High Growth Average (SD)	Low Growth Average (SD)	High Growth Average (SD)
Myristic acid (14:0)	0.91 (0.18)	0.91 (0.17)	0.84 (0.35)	0.85 (0.09)
Palmitic acid (16:0)	14.9 (1.00)	15.4 (1.51)	14.6 (1.77)	15.8 (2.23)
Palmitoleic acid (16:1w7)	1.80 (0.40)	1.50 (0.74)	2.27 (0.90)	2.08 (0.33)
Stearic acid (18:0)	12.1 ^a^ (0.66)	13.0 (0.93)	13.9 ^b^ (1.59)	13.9 (0.58)
Oleic acid (18:1w9)	35.2 ^a^ (2.56)	34.9 ^x^ (2.68)	42.3 ^b^ (4.10)	41.3 ^y^ (1.79)
Linoleic acid (18:2w6)	26.6 ^a^ (2.89)	24.3 ^x^ (2.27)	15.7 ^b^ (3.73)	14.6 ^y^ (2.17)
γ-linolenic acid (18:3w6)	0.28 ^a^ (0.27)	0.12 (0.04)	0.00 ^b,‡^ (0.00)	0.08 ^¶^ (0.05)
α-linolenic acid (18:3w3)	4.20 ^a^ (1.48)	3.40 ^x^ (0.54)	5.93 ^b^ (0.99)	6.42 ^y^ (0.79)
Stearidonic acid (18:4w3)	0.00 ^§^ (0.00)	0.13^¤^ (0.16)	0.00 ^‡^ (0.00)	0.17 ^¶^ (0.05)
Arachdic acid (20:0)	0.07 ^a,§^ (0.04)	0.35 ^x,¤^ (0.05)	0.17 ^b^ (0.07)	0.23 ^y^ (0.08)
Paullinic acid (20:1w7)	0.18 ^a,§^ (0.07)	0.57^¤^ (0.07)	0.29 ^b,‡^ (0.07)	0.63 ^¶^ (0.11)
Eicosenoic acid (20:2w6)	0.36 (0.08)	0.45 (0.14)	0.33 (0.08)	0.35 (0.04)
Mead acid (20:3w9)	0.10 ^a^ (0.04)	0.07 (0.03)	0.04 ^b,‡^ (0.03)	0.09 ^¶^ (0.02)
h-γ-linolenic acid (20:3w6)	0.27 ^§^ (0.06)	0.38 ^x,¤^ (0.08)	0.25 (0.04)	0.26 ^y^ (0.02)
Arachidonic acid (20:4w6)	1.79 ^§^ (0.34)	2.35 ^x,¤^ (0.30)	1.54 (0.29)	1.58 ^y^ (0.19)
Eicosatrienoic acid (20:3w3)	0.14 ^a^ (0.04)	0.16 ^x^ (0.03)	0.22 ^b^ (0.05)	0.25 ^y^ (0.07)
Eicosatetraenoic acid (20:4w3)	0.08 ^a,§^ (0.04)	0.00^¤^ (0.01)	0.28 ^b,‡^ (0.11)	0.01 ^¶^ (0.01)
Eicosapentaenoic acid (20:5w3)	0.13 ^§^ (0.03)	0.43 ^x,¤^ (0.10)	0.30 (0.18)	0.29 ^y^ (0.09)
Behenic acid (22:0)	0.34 (0.08)	0.36 ^x^ (0.04)	0.31 (0.07)	0.26 ^y^ (0.04)
Erucic acid (22:1w9)	0.00 ^§^ (0.00)	0.05^¤^ (0.05)	0.00 ^‡^ (0.00)	0.07 ^¶^ (0.03)
Docosatetraenoic (adrenic) acid (22:4w6)	0.04 ^§^ (0.05)	0.15 ^x,¤^ (0.05)	0.04 (0.05)	0.03 ^y^ (0.04)
Omega-6 docosapentaenoic acid (22:5w6)	0.07 ^a^ (0.11)	0.00 (0.00)	0.21 ^b,‡^ (0.05)	0.00 ^¶^ (0.00)
Omega-3 docosapentaenoic acid (22:5w3)	0.09 ^§^ (0.05)	0.18^¤^ (0.05)	0.13 ^‡^ (0.03)	0.22 ^¶^ (0.02)
Docosahexaenoic acid (22:6w3)	0.17 (0.04)	0.16 ^x^ (0.03)	0.17 ^‡^ (0.04)	0.10 ^y,¶^ (0.01)
Lignoceric acid (24:0)	0.00 ^§^ (0.00)	0.38 ^x,¤^ (0.06)	0.00 ^‡^ (0.00)	0.19 ^y,¶^ (0.02)
Nervonic acid (24:1)	0.16 ^§^ (0.07)	0.39^¤^ (0.39)	0.16 (0.18)	0.18 (0.07)
**Fatty Acid Group**	**Low Growth Average (SD)**	**High Growth Average (SD)**	**Low Growth Average (SD)**	**High Growth Average (SD)**
Saturates	28.3 ^a,§^ (0.99)	30.4^¤^ (1.08)	29.8 ^b^ (0.98)	31.3 (1.76)
Monoenes	35.6 ^a^ (2.64)	35.9 ^x^ (2.83)	42.8 ^b^ (4.25)	42.2 ^y^ (1.93)
Polyunsaturated fatty acids	34.3 ^a^ (3.07)	32.3 ^x^ (2.56)	25.2 ^b^ (4.62)	24.5 ^y^ (2.91)
Highly unsaturated fatty acids (HUFA)	2.88 ^§^ (0.39)	3.88 ^x,¤^ (0.48)	3.19 (0.54)	2.84 ^y^ (0.32)
Total omega-3 fatty acids	4.80 ^a^ (1.53)	4.45 ^x^ (0.44)	7.03 ^b^ (1.23)	7.45 ^y^ (0.91)
Total omega-6 fatty acids	29.4 ^a^ (3.23)	27.7 ^x^ (2.67)	18.1 ^b^ (3.85)	16.9 ^y^ (2.34)
Total omega-9 fatty acids	35.4 ^a^ (2.67)	35.3 ^x^ (2.81)	42.5 ^b^ (4.19)	41.6 ^y^ (1.86)
Omega-6/Omega-3	6.96 ^a^ (3.30)	6.29 ^x^ (0.94)	2.60 ^b^ (0.50)	2.28 ^y^ (0.29)
Omega 3 HUFA	21.3 ^a^ (3.90)	23.9 ^x^ (2.36)	34.2 ^b^ (3.75)	30.6 ^y^ (3.60)
Omega 6 HUFA	78.7 ^a^ (3.90)	76.1 ^x^ (2.36)	65.9 ^b^ (3.75)	69.4 ^y^ (3.60)

^1^ Differing superscripts (^a,b^) in averages columns are significantly different at (α = 0.05) for NC Zoo DBS Low Pasture Growth samples compared to Busch Gardens Tampa DBS Low Growth samples; differing superscripts (^x,y^) in averages columns are significantly different at (α = 0.05) for NC Zoo DBS High Pasture Growth samples compared to Busch Gardens Tampa DBS High Growth samples; differing superscripts (^§,¤^) in averages columns are significantly different at (α = 0.05) for NC Zoo DBS Low Pasture Growth samples compared to NC Zoo DBS High Growth samples; and differing superscripts (^‡,¶^) in averages columns are significantly different at (α = 0.05) for Busch Gardens Tampa DBS Low Pasture Growth samples compared to Busch Gardens Tampa DBS High Growth samples.

## Data Availability

The data that support the findings of this study are available from the corresponding author upon reasonable request.

## Supplementary Materials

The following are available online at https://www.mdpi.com/article/10.3390/ani11113063/s1. Table S1: Preliminary Fatty Acid (%) Profile Ranges of NC Zoo DBS Samples (*n* = 7) and Busch Gardens Tampa DBS Samples (*n* = 5) from Human Managed Southern White Rhinoceroses (*Ceratotherium simum simum*).
